# Supplementary material for: Buxus and Tetracentron genomes help resolve eudicot genome history
Source: Nat Commun. 2022 Feb 2;13:643. doi: 10.1038/s41467-022-28312-w (PMC8810787; doi:10.1038/s41467-022-28312-w)
Supplement: Supplementary file 1 — Supplementary Information [file 41467_2022_28312_MOESM1_ESM.pdf]

Buxus and *Tetracentron* genomes help resolve eudicot genome history

Chanderbali *et al*.

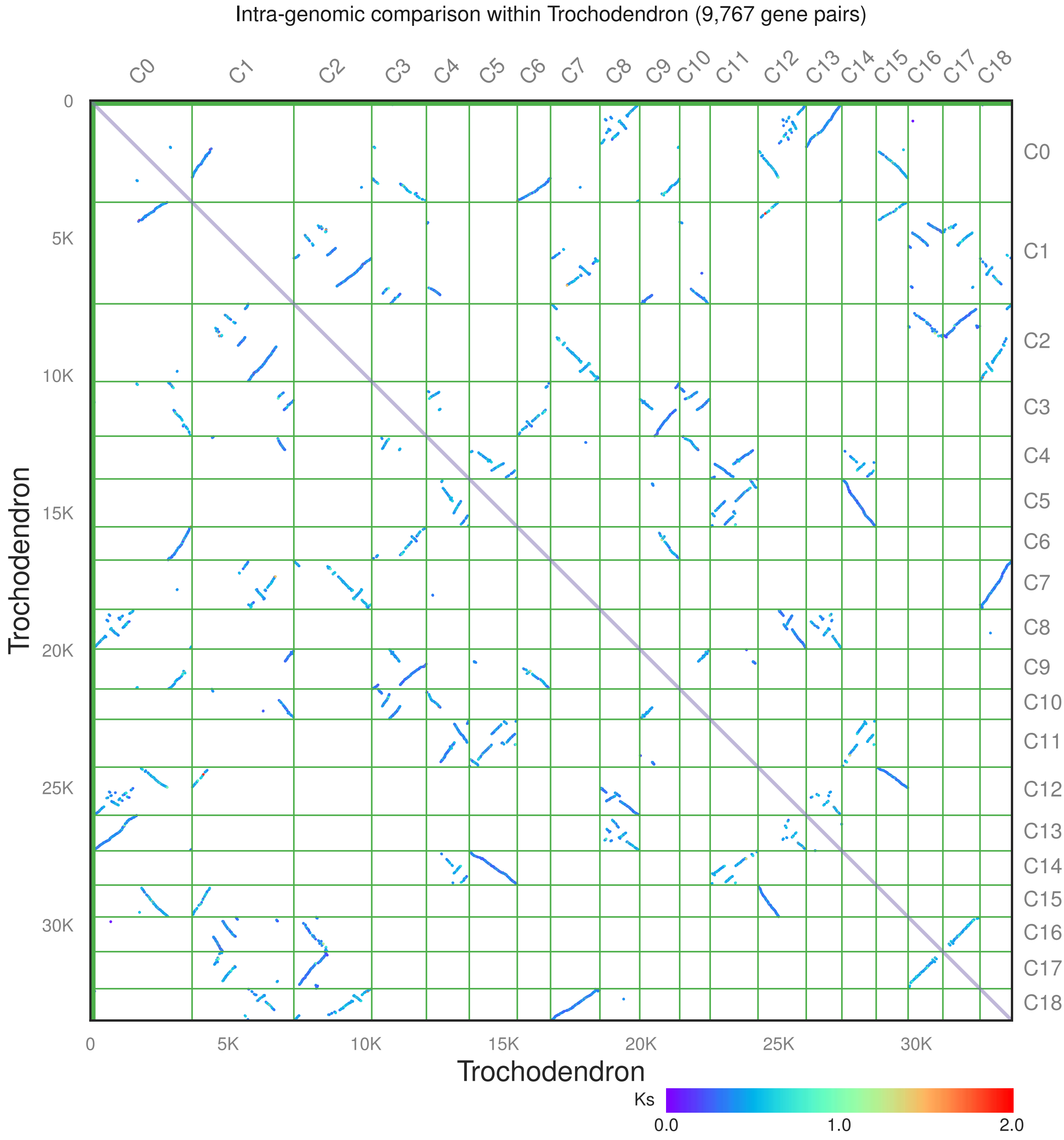

Supplementary Figure 1. Dotplot of intragenomic synteny in *Trochodendron aralioides*. Synteny blocks are colored by their *Ks* values according to the color scale. Source data are provided as a Source Data file.

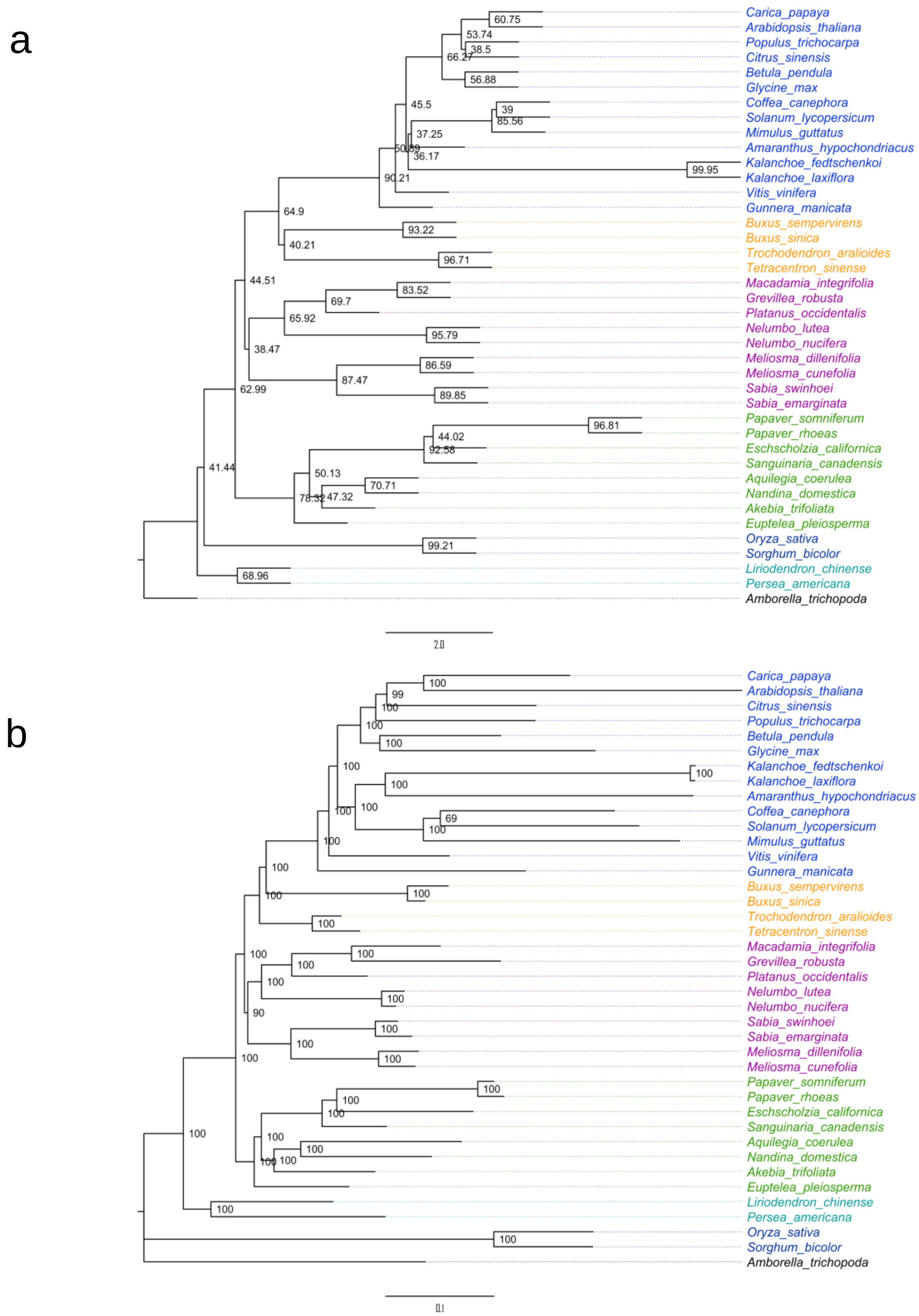

Supplementary Figure 2. Angiosperm phylogeny based on the Angiosperms353 loci emphasizing relations of early-diverging eudicots. Phylograms depict (a) the coalescent solution of individual RAxML gene trees and (b) partitioned RAxML analysis of a supermatrix of nucleotide sequence alignments. Node labels indicate quartet (a) and bootstrap support values (b).

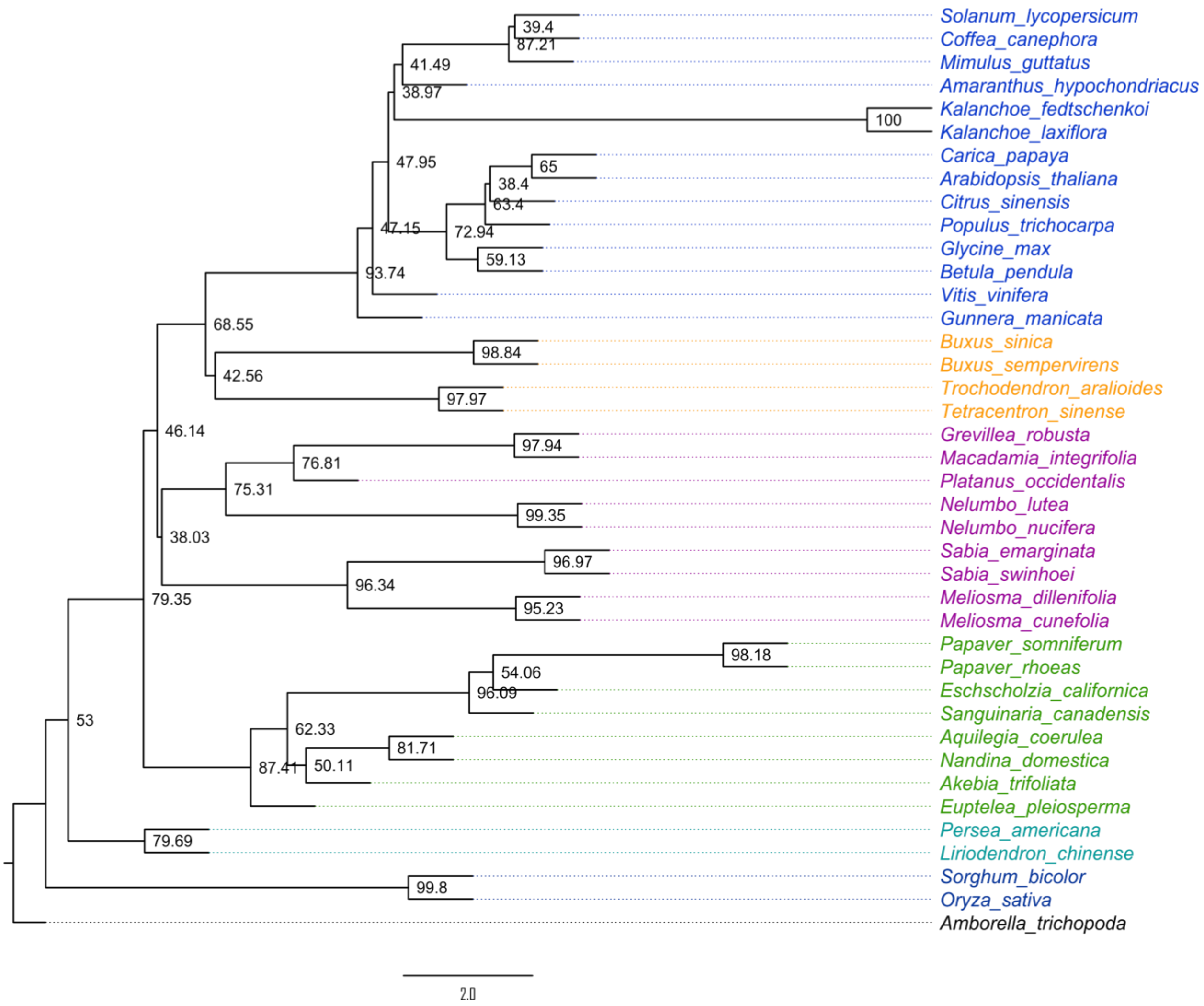

Supplementary Figure 3. Angiosperm phylogeny emphasizing relations of early-diverging eudicots produced by coalescence analysis of RAxML gene trees for 2573 orthogroups. Branch lengths are measured in coalescence units, and node labels indicate quartet support values.

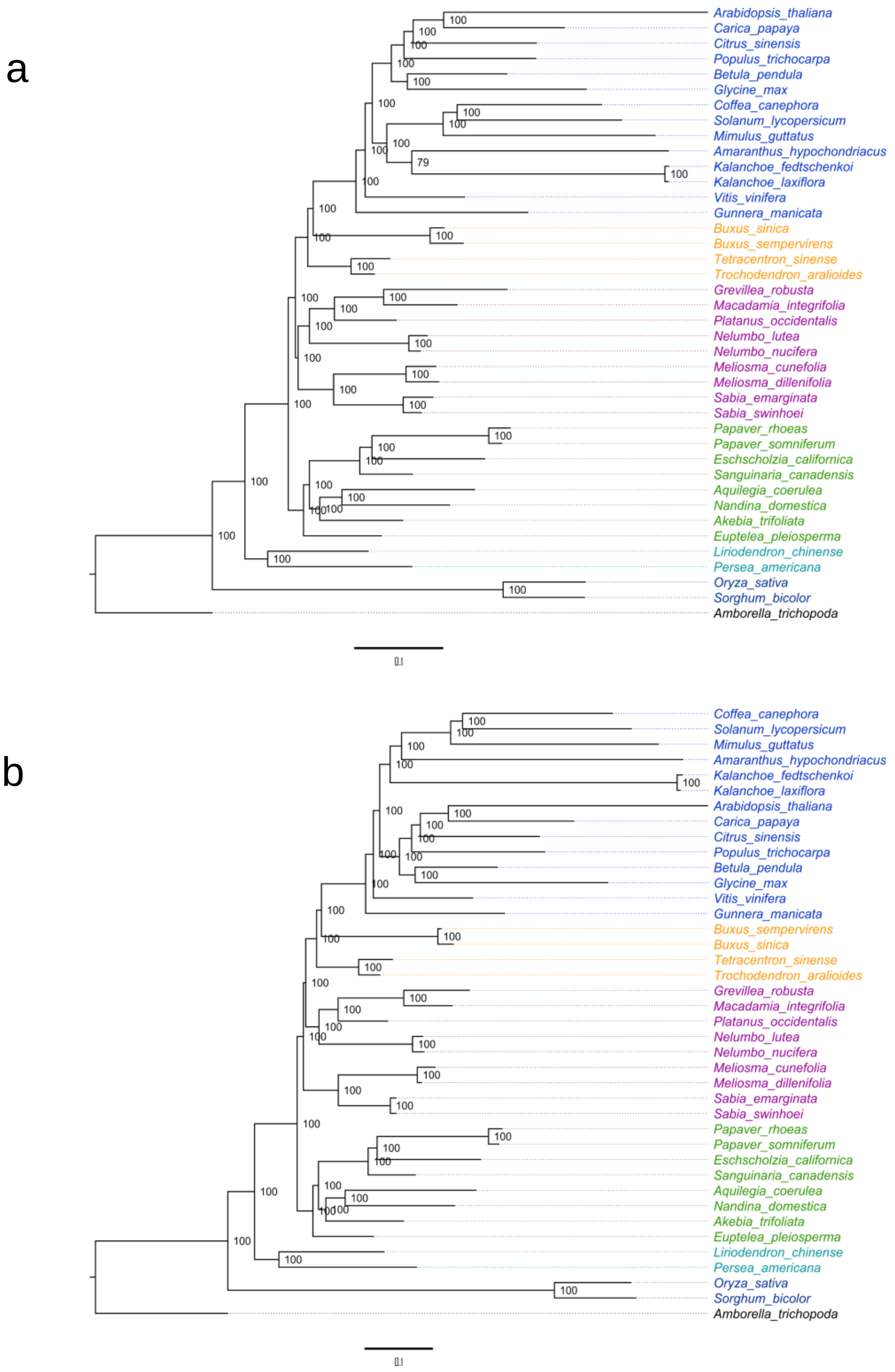

Supplementary Figure 4. Angiosperm phylogeny emphasizing relations of early-diverging eudicots produced by Bayesian inference. Phylograms depict consensus trees from partitioned analyses of supermatrices constructed from nucleotide alignments of the Angiosperms353 (a) and BUSCO (b) loci. Node values indicate posterior probability (PP) percentages.

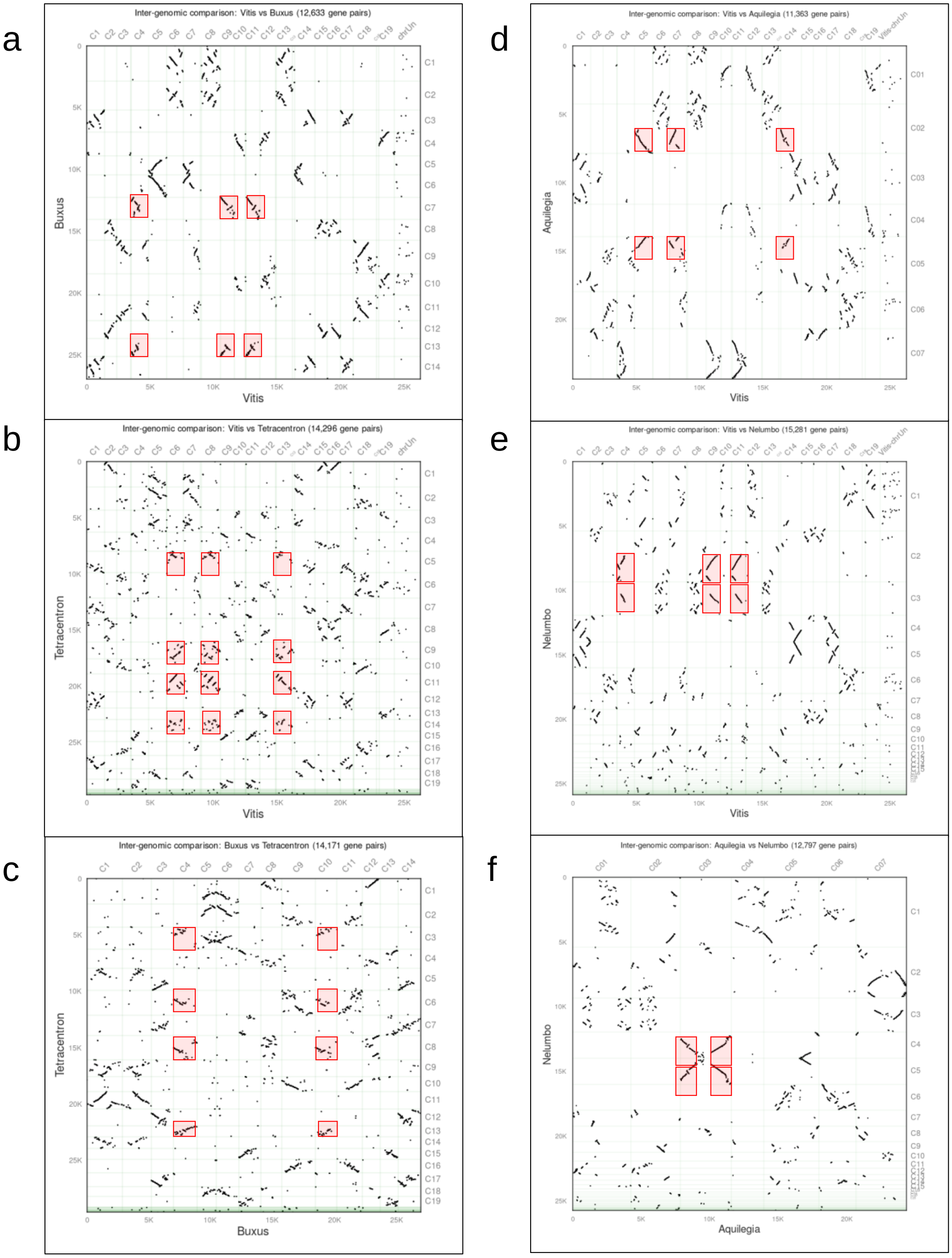

Supplementary Figure 5. Syntenic alignments of early-diverging and core eudicot genomes. a-c *Buxus sinica* and *Tetracentron sinense* versus *Vitis vinifera* and each other, respectively. d-f *Aquilegia coerulea* and *Nelumbo nucifera* versus *Vitis* and each other, respectively. Colored blocks in the plots highlight exemplar syntenic regions across genomes. Source data are provided as a Source Data file.

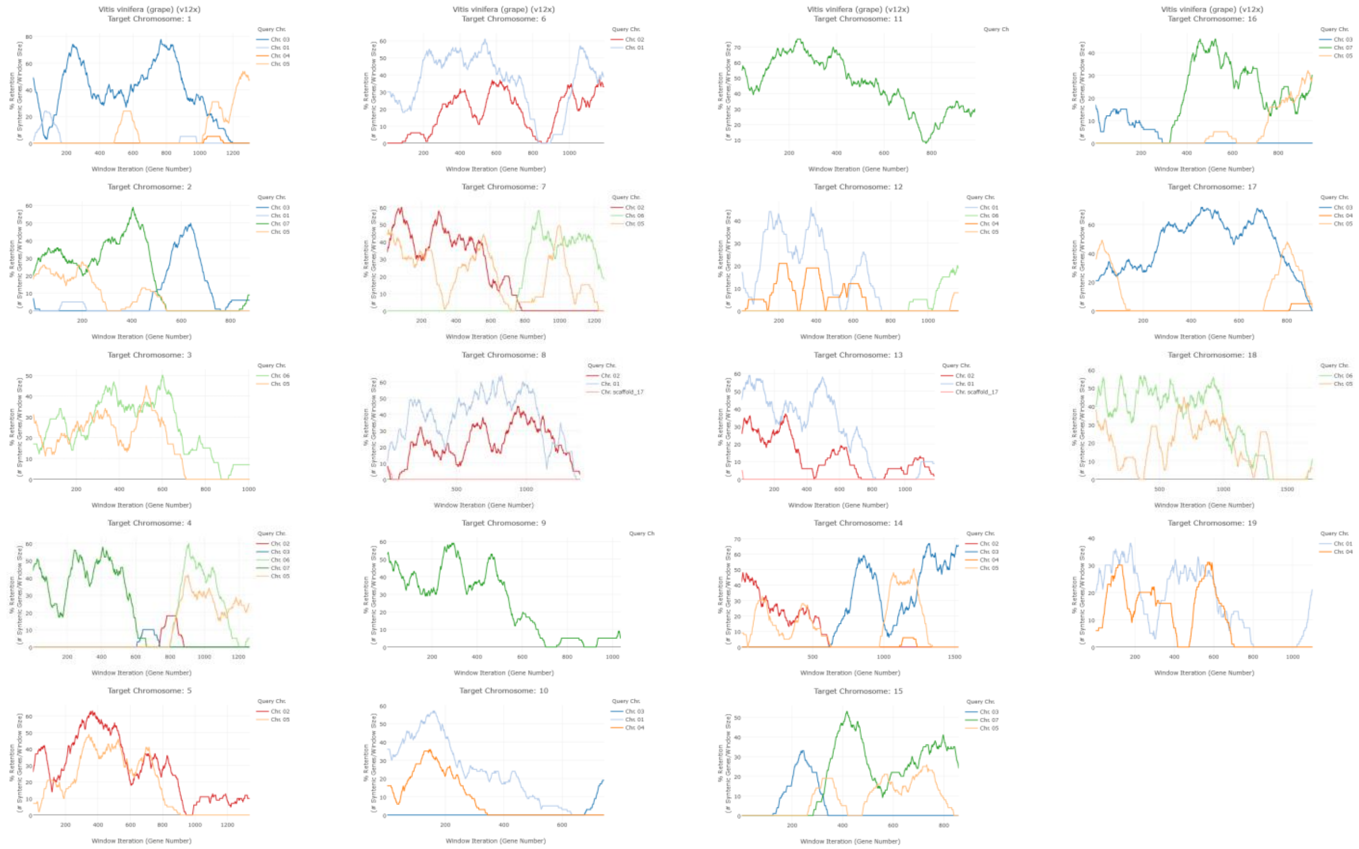

Supplementary Figure 6. FractBias plots comparing *Vitis vinifera* (target genome) with *Aquilegia coerulea*, (query genomes). Each item in the figure depicts a pair-wise comparison between one *Vitis* chromosome (1through 19) and the corresponding syntenic regions of the *Aquilegia* genome. X-axes correspond to iteratedsliding windows of 100 genes on the target chromosome. Y-axes indicate gene retention percentages at syntenic locations on query chromosomes. Plots can be regenerated at https://genomevolution.org/r/1apmi

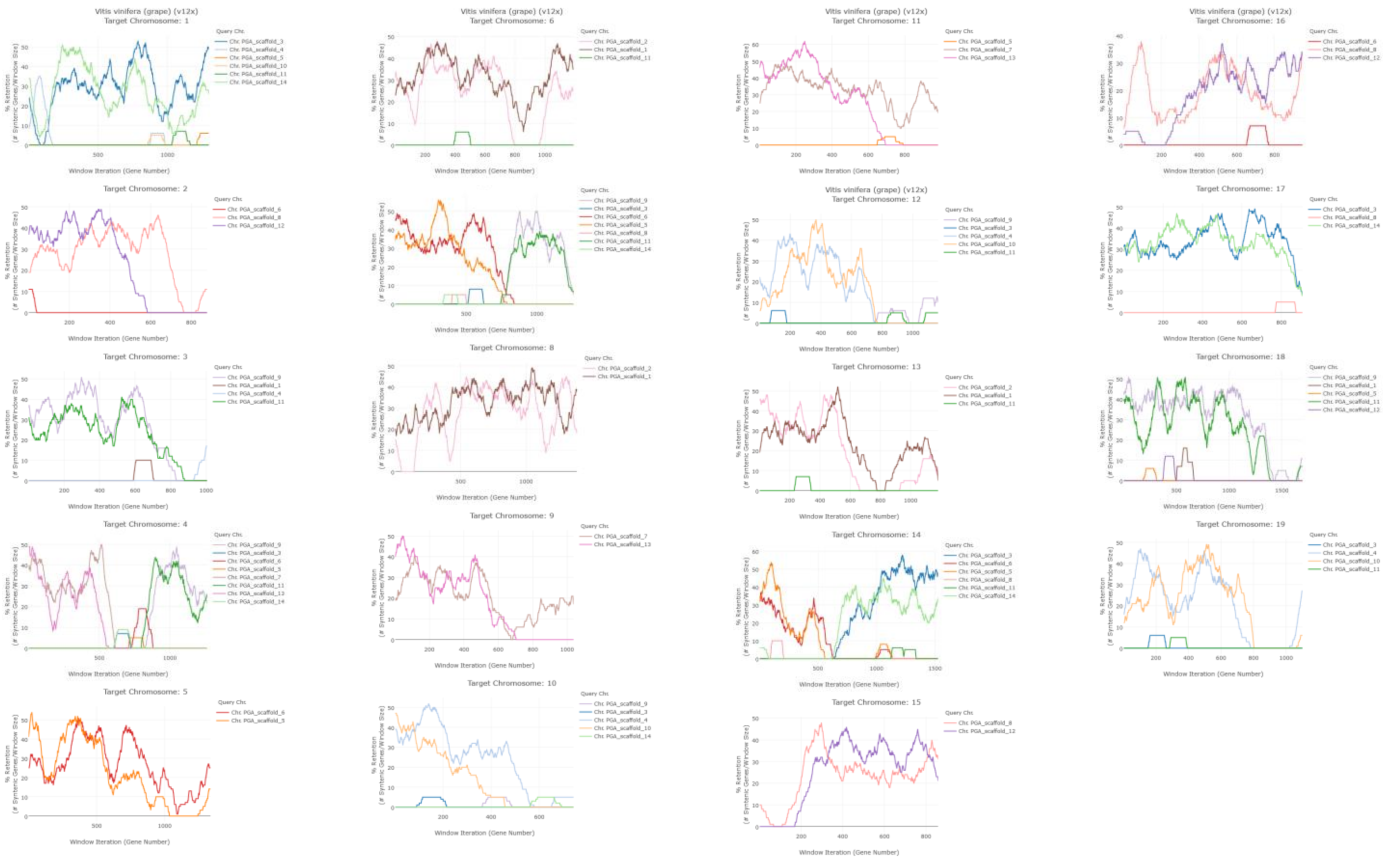

Supplementary Figure 7. FractBias plots comparing *Vitis vinifera* (target genome) with *Buxus sinica* (query genome). Each item in the figure depicts a pair-wise comparison between one *Vitis* chromosome (1 through 19) and the corresponding syntenic regions of the *Buxus* genome.X-axes correspond to iterated sliding windows of 100 genes on the target chromosome. Y-axes indicate gene retention percentages at syntenic locations on query chromosomes. Plots can be regenerated at https://genomevolution.org/r/1fksc.

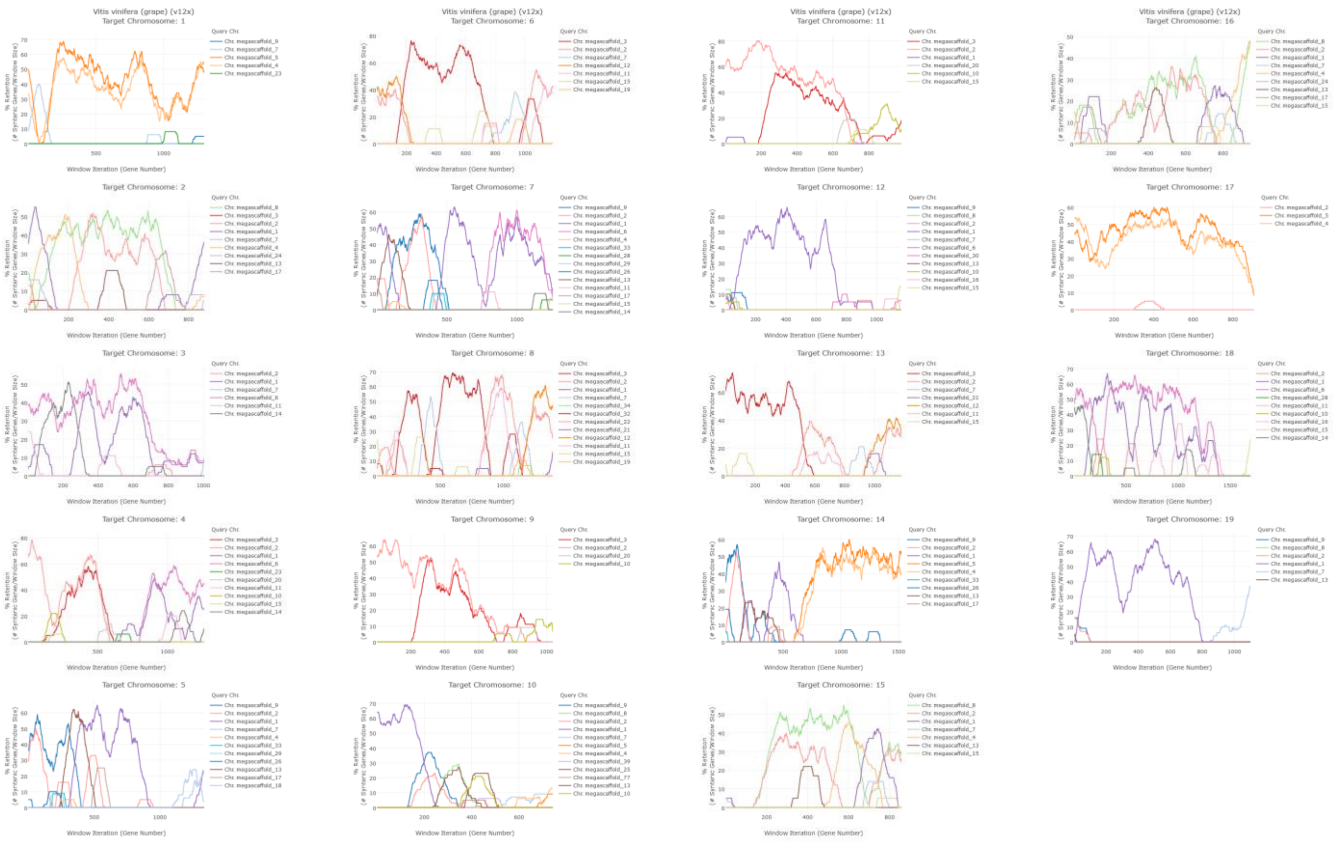

Supplementary Figure 8. FractBias plots comparing *Vitis vinifera* (target genome) with *Nelumbo nuicfera*(query genome). Each item in the figure depicts a pair-wise comparison between one *Vitis* chromosome (1 through 19) and the corresponding syntenic regions of the *Nelumbo* genome. X-axes correspond to iterated sliding windows of 100 genes on the target chromosome. Y-axes indicate gene retention percentages at syntenic locations on query chromosomes. Plots can be regenerated at https://genomevolution.org/r/1fkub

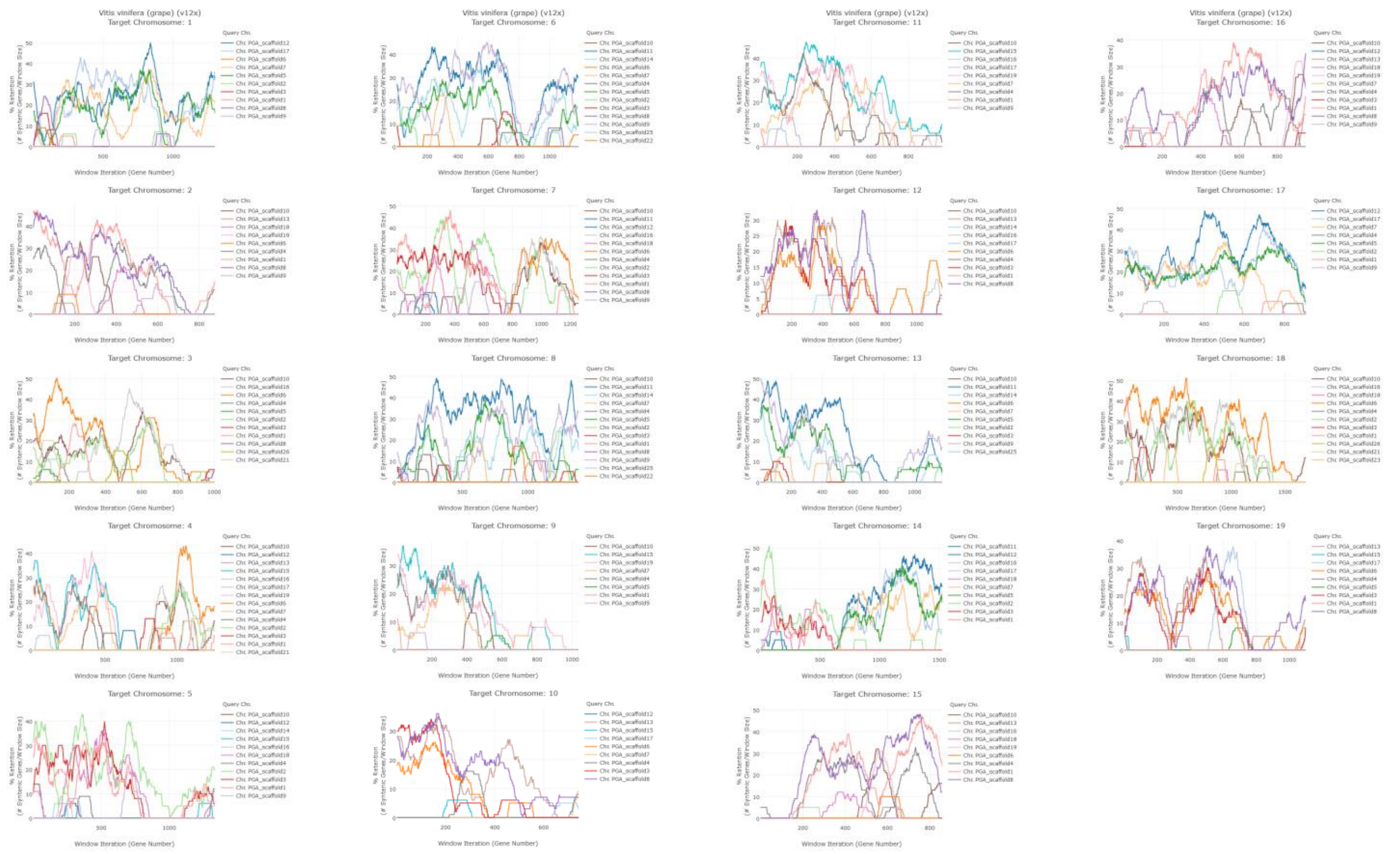

Supplementary Figure 9. FractBias plots comparing *Vitis vinifera* (target genome) with *Tetracentron sinense*(query genome). Each item in the figure depicts a pair-wise comparison between one *Vitis* chromosome (1 through 19) and the corresponding syntenic regions of the *Tetracentron* genome. X-axes correspond to iterated sliding windows of 100 genes on the target chromosome. Y-axes indicate gene retention percentages at syntenic locations on query chromosomes. Plots can be regenerated at https://genomevolution.org/r/1fkss

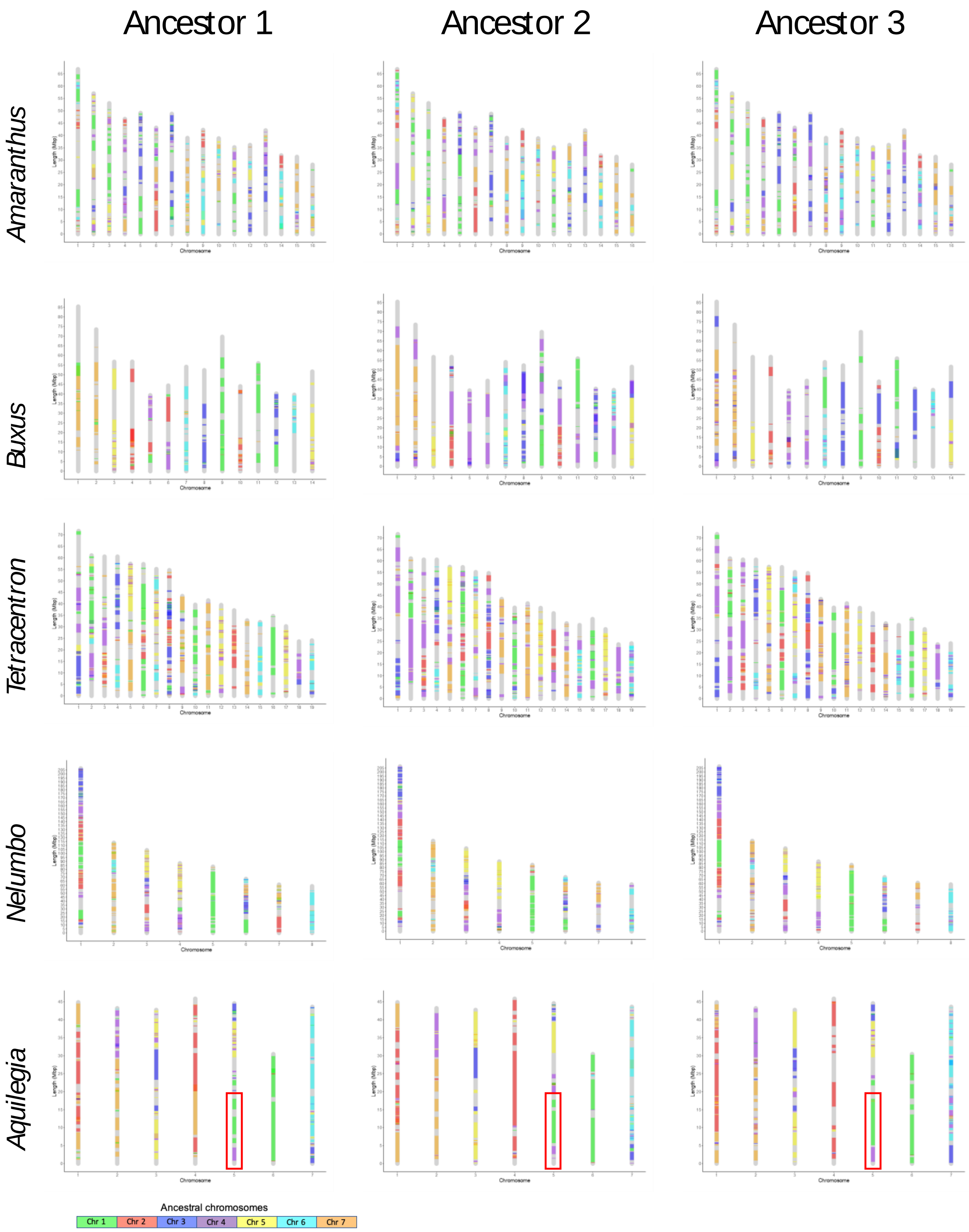

Supplementary Figure 10. Ancestral genomes mapped to the modern of genomes *Amaranthus, Buxus Tetracentron, Nelumbo, and Aquilegia.* Color scheme for ancestral chromosomes 1-7 is provided lower left. Source data are provided as a Source Data file.
