# Supplementary material for: Buxus and Tetracentron genomes help resolve eudicot genome history
Source: Nat Commun. 2022 Feb 2;13:643. doi: 10.1038/s41467-022-28312-w (PMC8810787; doi:10.1038/s41467-022-28312-w)
Supplement: Supplementary file 3 — Description of Additional Supplementary Files [file 41467_2022_28312_MOESM3_ESM.pdf]

Description of Additional Supplementary Files

Supplementary Data 1. Genome assembly metrics for *Buxus sinica* and *Tetracentron sinense*.

Supplementary Data 2. BUSCO assessment metrics for *Buxus sinica* and *Tetracentron sinense* genome assemblies and annotated gene models.

Supplementary Data 3. Repeat content of the *Buxus sinica* and *Tetracentron sinense* genome assemblies.

Supplementary Data 4. Clade memberships and their proportional representation among single copy gene trees.

Supplementary Data 5. Modern distribution and representation of each ancestral chromosome derived from macro- and microsynteny alignments.

Supplementary Data 6: Choppiness of painting on extant genomes.

Supplementary Data 7. Flow cytometric estimation of nuclear DNA content.

Supplementary Data 8. List of species and data sources.
