# Supplementary material for: Buxus and Tetracentron genomes help resolve eudicot genome history
Source: Nat Commun. 2022 Feb 2;13:643. doi: 10.1038/s41467-022-28312-w (PMC8810787; doi:10.1038/s41467-022-28312-w)
Supplement: Supplementary file 12 — Reporting Summary [file 41467_2022_28312_MOESM12_ESM.pdf]

nature portfolio

Corresponding author(s): Andre Chanderbali

Last updated by author(s): Dec 20, 2021

Reporting Summary

Nature Portfolio wishes to improve the reproducibility of the work that we publish. This form provides structure for consistency and transparency in reporting. For further information on Nature Portfolio policies, see our Editorial Policies and the Editorial Policy Checklist.

Statistics

For all statistical analyses, confirm that the following items are present in the figure legend, table legend, main text, or Methods section.

n/a	Confirmed	
[x]	[ ]	The exact sample size (*n*) for each experimental group/condition, given as a discrete number and unit of measurement
[x]	[ ]	A statement on whether measurements were taken from distinct samples or whether the same sample was measured repeatedly
[x]	[ ]	The statistical test(s) used AND whether they are one- or two-sided<br>*Only common tests should be described solely by name; describe more complex techniques in the Methods section.*
[x]	[ ]	A description of all covariates tested
[x]	[ ]	A description of any assumptions or corrections, such as tests of normality and adjustment for multiple comparisons
[x]	[ ]	A full description of the statistical parameters including central tendency (e.g. means) or other basic estimates (e.g. regression coefficient) AND variation (e.g. standard deviation) or associated estimates of uncertainty (e.g. confidence intervals)
[x]	[ ]	For null hypothesis testing, the test statistic (e.g. *F*, *t*, *r*) with confidence intervals, effect sizes, degrees of freedom and *P* value noted<br>*Give P values as exact values whenever suitable.*
[ ]	[x]	For Bayesian analysis, information on the choice of priors and Markov chain Monte Carlo settings
[x]	[ ]	For hierarchical and complex designs, identification of the appropriate level for tests and full reporting of outcomes
[x]	[ ]	Estimates of effect sizes (e.g. Cohen's *d*, Pearson's *r*), indicating how they were calculated

Our web collection on statistics for biologists contains articles on many of the points above.

Software and code

Policy information about availability of computer code

Data collection	No software was used for data collection
Data analysis	Genome assembly: pb-assembly v0.0.6; FALCON/FALCON-Unzip v0.3.1, Purge Haplotigs v1.0.4<br>Genome annotation: MAKER v3.01.03; LTRharvest (GenomeTools) v1.5.9 ; LTRdigest (GenomeTools) v1.5.9; MITE-Hunter v20111101; RepeatModeler v1.0.8; RepeatMasker v4.1.1; Augustus v3.4.0; SNAP v0.15.4<br>Transcriptome assembly: Trinity v2.12.0; Trimmomatic v0.39; TransDecoder v5.0.2<br>Phylogenetic analyses: RAxML v8.2.12; Astral-Pro v1.1.3; IQ-TREE v2.1.0; BLAST v2.10.1; MAFFT v7.402; PAL2NAL v14; trimAl v1.2; BUSCO v4.1.4; Orthofinder v2.5.2; FASCONCAT-G v1.02<br>Synteny analyses: MCscan in JCVI utility libraries v1.0.13; CoGe platform (https://genomevolution.org).<br>Ancestral genome: RACCROCHE (https://github.com/jin-repo/RACCROCHE).<br>Flow Cytometry: BD CellQuest Pro v6.0

For manuscripts utilizing custom algorithms or software that are central to the research but not yet described in published literature, software must be made available to editors and reviewers. We strongly encourage code deposition in a community repository (e.g. GitHub). See the Nature Portfolio guidelines for submitting code & software for further information.

Data

Policy information about availability of data

All manuscripts must include a data availability statement. This statement should provide the following information, where applicable:

- Accession codes, unique identifiers, or web links for publicly available datasets
- A description of any restrictions on data availability
- For clinical datasets or third party data, please ensure that the statement adheres to our policy

The following Data Availability statement is included:
All raw sequence reads generated in this study have been deposited in the NCBI database under accession numbers PRJNA549075 (https://www.ncbi.nlm.nih.gov/bioproject/549075), PRJNA547721 (https://www.ncbi.nlm.nih.gov/bioproject/547721), and PRJNA548936 (https://www.ncbi.nlm.nih.gov/bioproject/548936). Additionally, the Buxus and Tetracentron genome assemblies, associated annotation files, and predicted CDS and protein sequences, along with all phylogenetic data sets analyzed here, and ancestral genome reconstructions have been deposited in the Dryad Digital Repository (https://doi.org/10.5061/dryad.cjsxksn6d). Source data are provided with this paper.

Field-specific reporting

Please select the one below that is the best fit for your research. If you are not sure, read the appropriate sections before making your selection.

[x] Life sciences [ ] Behavioural & social sciences [ ] Ecological, evolutionary & environmental sciences

For a reference copy of the document with all sections, see nature.com/documents/nr-reporting-summary-flat.pdf

Life sciences study design

All studies must disclose on these points even when the disclosure is negative.

Sample size	Single individuals of Buxus sinica and Tetracentron sinense were sampled for genome sequencing and transcriptome sequencing. Single individuals of Buxus sempervirens, Gunnera manicata, Meliosma dillenifolia, Nelumbo lutea, Sabia emarginata, Sabia swinhonei, and Trochodendron aralioides were sampled for transcriptome sequencing. Sampling was restricted to single individuals to avoid mixing different genotypes in genome and transcriptome assemblies.
Data exclusions	Pacbio reads shorter that 5000 bases were excluded prior to genome assembly. Adaptor sequences and low-quality base calls were trimmed from Illumina reads prior to transcriptome assemblies. Phylogenetic data sets were trimmed to discard poorly aligned sequences and residues, and alignments with fewer than four sequences and without Buxales or Trochdendrales were excluded.
Replication	Bootstrap analyses with 1000 replicates were used in Maximum Likelihood phylogenetic analyses. Two independent parallel runs of four Metropolis-coupled Monte Carlo Markov Chains were run for 10 million generations with sampling every 1000 generations in Bayesian Inference phylogenetic analyses. Both strategies indicate strong statistical support for the respective findings.
Randomization	Randomization was not needed because a single sample was used to represent each species. Maximum Likelihood and Bayesian Inference phylogenetic analyses incorporate randomization in their respective workflow tasks.
Blinding	The study did not involve experimental manipulation. As such, blinding was not part of the research strategy.

Reporting for specific materials, systems and methods

We require information from authors about some types of materials, experimental systems and methods used in many studies. Here, indicate whether each material, system or method listed is relevant to your study. If you are not sure if a list item applies to your research, read the appropriate section before selecting a response.

Materials & experimental systems

n/a	Involved in the study	
[x]	[ ]	Antibodies
[x]	[ ]	Eukaryotic cell lines
[x]	[ ]	Palaeontology and archaeology
[x]	[ ]	Animals and other organisms
[x]	[ ]	Human research participants
[x]	[ ]	Clinical data
[x]	[ ]	Dual use research of concern

Methods

n/a	Involved in the study	
[x]	[ ]	ChIP-seq
[ ]	[x]	Flow cytometry
[x]	[ ]	MRI-based neuroimaging

Flow Cytometry

Plots

Confirm that:

- [ ] The axis labels state the marker and fluorochrome used (e.g. CD4-FITC).
- [ ] The axis scales are clearly visible. Include numbers along axes only for bottom left plot of group (a 'group' is an analysis of identical markers).
- [ ] All plots are contour plots with outliers or pseudocolor plots.
- [ ] A numerical value for number of cells or percentage (with statistics) is provided.

Methodology

Sample preparation	Flow cytometry was performed as a paid service by the Benaroya Research Institute (Seattle, WA). Fresh leaf tissues were shipped on ice to the service provider.
Instrument	FACScalibur flow cytometer
Software	CellQuest software v6.0
Cell population abundance	Flow cytometry was used for genome size estimation purposes. No post-sorting fraction was collected.
Gating strategy	Unsure of the gating strategy applied by the service provider.

- [ ] Tick this box to confirm that a figure exemplifying the gating strategy is provided in the Supplementary Information.
